# Systematic approach to Escherichia coli cell population control using a genetic lysis circuit

**DOI:** 10.1186/1752-0509-8-S5-S7

**Published:** 2014-12-12

**Authors:** Chih-Yuan Hsu, Tsu-Chun Yu, Ling-Jiun Lin, Rei-Hsing Hu, Bor-Sen Chen

**Affiliations:** 1 Lab of Control and Systems Biology, Department of Electrical Engineering, National Tsing Hua University, Hsinchu, 30013, Taiwan; 2 Lab of Genetic Circuit Design, Department of Electrical Engineering, National Tsing Hua University, Hsinchu, 30013, Taiwan

**Keywords:** Genetic lysis circuit, Cell population control, Promoter-RBS library

## Abstract

**Background:**

Cell population control allows for the maintenance of a specific cell population density. In this study, we use lysis gene BBa_K117000 from the Registry of Standard Biological Parts, formed by MIT, to lyse *Escherichia coli *(*E. coli*). The lysis gene is regulated by a synthetic genetic lysis circuit, using an inducer-regulated promoter-RBS component. To make the design more easily, it is necessary to provide a systematic approach for a genetic lysis circuit to achieve control of cell population density.

**Results:**

Firstly, the lytic ability of the constructed genetic lysis circuit is described by the relationship between the promoter-RBS components and inducer concentration in a steady state model. Then, three types of promoter-RBS libraries are established. Finally, according to design specifications, a systematic design approach is proposed to provide synthetic biologists with a prescribed I/O response by selecting proper promoter-RBS component set in combination with suitable inducer concentrations, within a feasible range.

**Conclusion:**

This study provides an important systematic design method for the development of next-generation synthetic gene circuits, from component library construction to genetic circuit assembly. In future, when libraries are more complete, more precise cell density control can be achieved.

## Background

Cell population control is a method of regulating cell density to maintain a self-sustaining population. Self-regulating mechanisms of cell population control have existed in nature for a long time. For example, the spore-forming bacterium *Bacillus subtilis *delays sporulation under nutrient-limited conditions by killing non-sporulating siblings and feeding on the dead cells to support spore formation [1]. Colicin-producing bacteria produce bacteriocins to destroy nearby competitors through amino-acid starvation or DNA damage [2]. Recently, a programmed control of cell population is proposed, using an endogenous genetic regulatory circuit [3]. However, to make genetic lysis circuit design more easily, it is necessary to provide a systematic approach to achieve control of cell population density.

Naturally occurring lytic systems have the ability to trigger host cell lysis with specific proteins under certain circumstances, such as in the presence of antibiotics or competitors, or under conditions of amino-acid starvation. Some species of bacteria and bacteriophage have lytic protein that induces cell lysis. For example, expression of cloned T4 phage genes *e *or *t *can be used to the disrupt *E. coli *cells [4,5]. Lysis by the T4 phage usually requires two gene products, transcribed by genes *e *and *t*, respectively. Gene *e *encodes a lysozyme, Gpe; gene *t *encodes a holin, Gpt. The expression of gene *e *weakens *E. coli *cell wall but does not lead to cell disruption, while the expression of gene *t *enables to induce cell lysis. Another example of a lytic protein is colicin E7 (ColE7), which is encoded by *E. coli*. Expression of ColE7 is regulated by an SOS response operon. The SOS response operon is composed of the *ceaE7, ceiE7 *and *celE7 *genes, the products of which are ColE7, ImE7 and LysE7, respectively [6]. ColE7 can be neutralized through the formation of a protein complex with the immunity protein ImE7. The ColE7-ImE7 complex is exported from the colicin-producing cells by the lysis protein LysE7, which induces lysis of the host cell through break down of the cell membrane [7-12].

Lysis protein is able to activate the outer membrane phospholipase A (OMPLA), which allows colicin to cross the cell envelope and enter into the medium. Colicins are plasmid-encoded bacteriocins produced by *E. coli *that have antibiotic-like activity against closely related bacteria [12,13]. Release of lysis protein can therefore control the population density of *E. coli*. In this study, lysis protein expression level in the genetic lysis circuit relies on tunable genetic components and inducer concentrations. The degree of cell lysis could therefore be fine-tuned by changing external inducer signals. Different inducer concentrations could result in different fates of the cells, for example severe death, modulated death, or slow growth. To make the design more easily, the lytic ability of the constructed genetic lysis circuit is described by the relationship between the promoter-RBS components and inducer concentrations in a steady state model. According to user-oriented specifications, the genetic lysis circuit can be constructed via selecting adequate promoter-RBS components in combination with a feasible range of inducer concentrations. In general, it requires long computation times when component libraries become large. Hence, a genetic algorithm (GA) search method is proposed to save time in evaluating and selecting promoter-RBS components.

This study provides an important systematic design method for the development of next-generation synthetic biology, from component library construction to genetic circuit assembly. When libraries are more complete, more precise cell density control for genetic lysis circuit can be achieved. The mechanism of cell lysis can be used to release useful macromolecules that cannot pass through cell membranes [4,14]. For example, an operon encoding *bcsA, bcsB, bcsC*, and *bcsD *is required for bacterial cellulose synthesis (bcs) in *Acetobacter xylinum *[15]. The expression of the operon can transform redundant glucose into cellulose to maintain intestinal peristalsis. In addition, an appropriate amount of cellulose has a proven role in preventing obesity. Cellulose, however, is a macromolecule that normally cannot pass through the cell membrane. A genetic lysis circuit can be combined with a bacterial cellulose synthesis system to promote cellulose release. In future, genetic lysis circuits may apply to development in, for example, drug discovery, metabolic control, and therapeutic treatment, with the help of the proposed design methodology.

The contributions of this paper are threefold: (1) Based on promoter-RBS kinetic strengths, we establish three kinds of promoter-RBS libraries. (2) Inducible promoter-RBS components are used to construct genetic lysis circuits with different lytic abilities for cell population control. (3) The proposed GA-based systematic searching method could provide synthetic biologists with a useful tool to design synthetic genetic lysis circuits.

## Methods

### Construction of genetic lysis circuit

**Figure 1:**
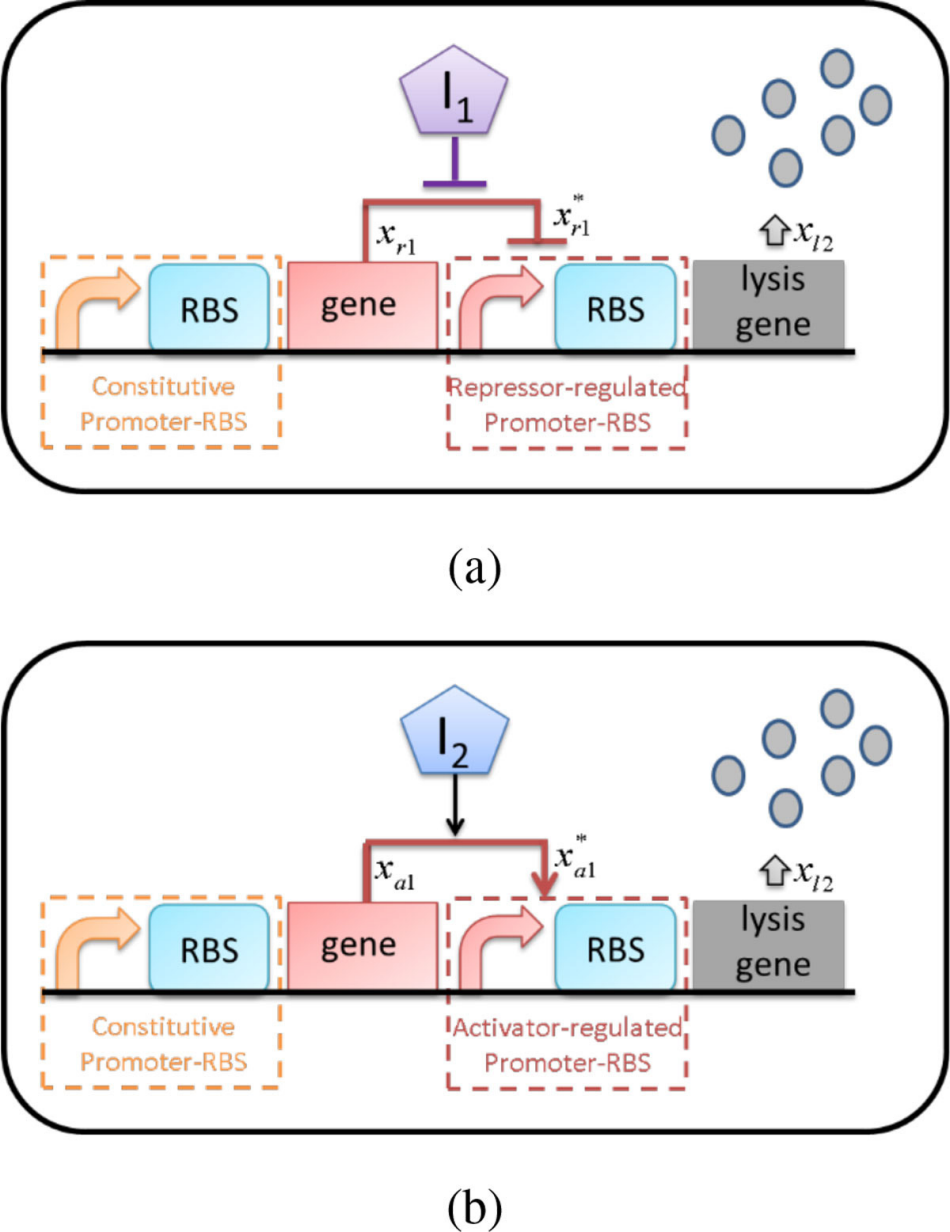
**Genetic lysis circuit for controlling cell population density**. (a) Inducible repressor-regulated circuit with lysis gene in *E. coli*. (b) Inducible activator-regulated circuit with lysis gene in *E. coli*.

Two type of synthetic genetic lysis circuits are shown in Figure 1(a) and (b), with different external inducers *I*_1 _and *I*_2_. In Figure 1(a), a constitutive promoter continuously produces the repressor protein *x*_r1_. The protein *x*_r1_represses the downstream promoter and reduces expression of the lysis gene. The inducer *I*_1 _can bind to the repressor protein and prevent binding to the promoter operon, enhancing expression of the lysis gene. In Figure 1(b), a constitutive promoter continuously produces the activator protein *x*_a1_. The protein *x*_a1_, however, needs to form a complex with the inducer *I*_2_. The complex constitutes a quorum sensing mechanism. When this complex accumulates, it activates the downstream promoter and enhances expression of the lysis gene.

### Dynamic model of genetic lysis circuit

Before we introduce the dynamic model of a genetic lysis circuit, a normal equation for cell growth, without the lysis protein, is as follows:

(1) $$Ṅ\left(t\right)=k\cdot N\left(t\right)\left(1-\frac{N\left(t\right)}{N_{\text{max}}}\right)$$

where *N *and *N_max _*denote cell population density (O.D. 600) and maximum cell population density, respectively, and *k *denotes the dilution rate due to cell growth. Since our study utilizes the lysis protein to regulate cell population density, the relationship between cell density and the concentration of the lysis protein can be described in the following equation:

(2) $$Ṅ\left(t\right)=k\cdot N\left(t\right)\left(1-\frac{N\left(t\right)}{N_{\text{max}}}\right)-\gamma_{N}\cdot N\left(t\right)\cdot x_{l}\left(t\right)$$

where *x_1 _*and *γ_N _*denote the concentration of the lysis protein and the lysis rate of the lysis protein, respectively. Under normal growth conditions, cells do not suffer from the toxicity of the lysis protein. We used the endogenous gene (BBa_K117000) encoded in *E. coli *to control cell population density. The lysis protein can activate OMPLA to cause cell lysis and reduce cell density [12,13]. From equation (2), it can be seen that the concentration of lysis protein *x_1 _*is the negative regulator of cell population density. Because lysis ability is related to the concentration of lysis protein *x_1 _*and the toxicity *γ_N _*of the lysis protein, we construct a dynamic model of the genetic lysis circuit to regulate the concentration of lysis protein *x_1_*, and thus lysis ability.

To construct the dynamic model, promoter-RBS regulation must be introduced. We first define the promoter-RBS regulation function *P*(*P_u_, P_1_, TF, I*), in which *P_u _*and *P_1 _*denote the maximum and minimum promoter-RBS strengths, respectively, *TF *denotes transcription factor concentration, and *I *denotes inducer concentration. This function describes the biochemical aspect of the transcription and translation process. The details of the function (the mathematical model) can be found in Additional file 1. The dynamic model of the genetic lysis circuit with the repressor-regulated promoter-RBS component in Figure 1(a) thus is described as follows:

(3) $$\begin{array}{c} \left\{\begin{array}{c} {ẋ}_{r1}\left(t\right)=P_{c1}\left(P_{u,i},0,0,0\right)-\left(\gamma_{x_{r1}}+k\right)\cdot x_{r1}\left(t\right)+\omega_{1}\left(t\right),\;\;\;i=1,...,C\\ {ẋ}_{l2}\left(t\right)=P_{r2}\left(P_{u,j},P_{l,j},x_{r1},I_{1}\right)-\left(\gamma_{x_{l2}}+k\right)\cdot x_{l2}\left(t\right)+\omega_{2}\left(t\right),\;j=1,...,R\\ Ṅ\left(t\right)=k\cdot N\left(t\right)\left(1-N\left(t\right)/N_{\text{max}}\right)-\gamma_{N}\cdot N\left(t\right)\cdot x_{l2}\left(t\right)+\omega_{3}\left(t\right) \end{array}\right)\\ P_{r2}\left(P_{u,j},P_{l,j},x_{r1},I_{1}\right)=P_{l,j}+\frac{P_{u,j}-P_{l,j}}{1+{\left(\frac{x_{r1}^{*}\left(x_{r1},I_{1}\right)}{K_{r2}}\right)}^{n_{r2}}},\;x_{r1}^{*}\left(x_{r1},I_{1}\right)=\frac{x_{r1}}{1+\left(\frac{I_{1}}{K_{I_{1}}}\right)} \end{array}$$

where (*i, j*) denote the *i^th ^*constitutive promoter-RBS component and the *j^th ^*repressor-regulated promoter-RBS component, respectively. *P*_c1 _(*P_u,j_*,0,0,0) denotes the promoter-RBS regulation activity of the first stage of the constitutive promoter-RBS components. *P_u,i _*denotes the maximum promoter-RBS strength of the *i^th ^*constitutive promoter-RBS component. *P*_r2_(*P*_u,j_,P_i,j_,x_r1_,I_1_) denotes the promoter-RBS regulation activity of the second stage of the repressor-regulated components. *P_u,j _*and *P_i,j _*denote the maximum and minimum promoter-RBS strengths of the *j^th ^*repressor-regulated promoter-RBS component, respectively. *I*_1 _denotes the concentration, and *x*_r1 _denotes the concentration of the repressor. *K*_r2 _and *n*_r2 _denote the binding affinity and binding cooperativity between the repressor $x_{r1}^{*}$ and the corresponding promoter-RBS component in the second stage, respectively. *K*_I1 _denotes the dissociation rate between the inducer *I*_1 _and the repressor *x*_r1_. *γ_N _*is the lysis rate of the lysis protein. *ω*_1_(*t*) and *ω*_2_(*t*) denote cellular noise in the transcriptional and translational processes, respectively. Finally, *ω*_3_(*t*) denotes cellular noise in the cell population density.

From equation (3), the change in concentration of repressor protein *x_r1 _*is due to the difference between the protein generation rate *P*_c1_(*P_u,j_*,0,0,0) and the protein degradation rate *γ*_xr1_, in combination with the dilution rate *k*. The repressor protein *x*_r1 _binds to the second stage of repressor-regulated components and reduces the regulation activity *P*_r2 _(*P_u,j_,P_i,j_,x_r1_*,I_1_). The inducer *I*_1 _can remove the inhibitory effect of the repressor and increase the regulation activity *P*_r2 _(*P*_u,j_,P_i,j_,x_r1_,I_1_). Similarly, the change in concentration of lysis protein *x*_l2 _is due to the difference between the protein generation rate *P*_r2 _(*P*_u,j_,P_i,j_,x_r1_,I_1_) and the protein degradation rate *γ*_xl2_, in combination with the dilution rate *k*. Cell population density is regulated by the concentration of lysis protein *x*_l2_. Lytic ability is therefore controlled by the regulation activity *P*_r2_(*P*_u,j_,P_i,j_,x_r1_,I_1_), and the four regulated factors *P*_u,j_, *P*_i,j_, *x*_r1_, and *I*_1 _can be used to control cell population density by selecting appropriate promoter-RBS components and inducer concentrations.

The only difference between Figures 1(a) and (b) is the replacement of a repressor-regulated promoter-RBS component with an activator-regulated promoter-RBS component. The dynamic model of the genetic lysis circuit in Figure 1(b) can be described as follows:

(4) $$\begin{array}{c} \left\{\begin{array}{c} {ẋ}_{a1}\left(t\right)=P_{c1}\left(P_{u,i},0,0,0\right)-\left(\gamma_{x_{a1}}+k\right)\cdot x_{a1}\left(t\right)+\omega_{1}\left(t\right),\;\;\;i=1,...,C\\ {ẋ}_{l2}\left(t\right)=P_{a2}\left(P_{u,m},P_{l,m},x_{a1},I_{2}\right)-\left(\gamma_{x_{l2}}+k\right)\cdot x_{l2}\left(t\right)+\omega_{2}\left(t\right),\;m=1,...,A\\ Ṅ\left(t\right)=k\cdot N\left(t\right)\left(1-N\left(t\right)/N\left(t\right)N_{\text{max}}\right)-\gamma_{N}\cdot N\left(t\right)\cdot x_{l2}\left(t\right)+\omega_{3}\left(t\right) \end{array}\right)\\ P_{a2}\left(P_{u,m},P_{l,m},x_{a1},I_{2}\right)=P_{l,m}+\frac{P_{u,m}-P_{l,m}}{1+{\left(\frac{K_{a2}}{x_{a1}^{*}\left(x_{a},I_{2}\right)}\right)}^{n_{a2}}},\;x_{a1}^{*}\left(x_{a1},I_{2}\right)=\frac{x_{a1}}{1+\left(\frac{K_{I_{2}}}{I_{2}}\right)} \end{array}$$

where (*i, m*) denote the *i^th ^*constitutive promoter-RBS component and the *m^th ^*activator-regulated promoter-RBS component, respectively. *I*_2 _denotes the concentration of the inducer, *x*_a1 _denotes the concentration of the activator protein, and *K*_a2 _and *n*_a2 _denote the binding affinity and the binding cooperativity between activator $x_{a1}^{*}$ and the corresponding promoter-RBS component in the second stage, respectively. Finally, *K*_I2 _denotes the dissociation rate between inducer *I*_2 _and activator *x*_a1_.

The dynamic models for the genetic lysis circuit are then transformed into steady-state models by assuming the derivatives of the dynamic models in (3) and (4) are equal to zero. The steady-state concentrations *x*_r1SS_, *x*_a1SS_, and *x*_l2SS _of the repressor protein, activator protein, and lysis protein, respectively, as well as the steady state cell population density *N_SS _*are obtained as follows:

(5) $$\left\{\begin{array}{c} x_{r1ss}=\frac{P_{c1}\left(P_{u,i},0,0,0\right)}{\gamma_{x_{r1}}+k}+v_{1},\;\;\;\;i=1,...,C\\ x_{l2ss}=\frac{P_{r2}\left(P_{u,j},P_{l,j},x_{r1ss},I_{1}\right)}{\gamma_{x_{l2}}+k}+v_{2},\;j=1,...,R\\ N_{ss}=N_{\text{max}}\left(1-\frac{\gamma_{N}}{k}{x_{l2}}_{ss}\right)+v_{3} \end{array}\right)$$

for the genetic lysis circuit with repressor-regulated promoter-RBS component in Figure 1(a), and

(6) $$\left\{\begin{array}{c} x_{a1ss}=\frac{P_{c1}\left(P_{u,i},0,0,0\right)}{\gamma_{x_{a1}}+k}+v_{1},\;\;\;\;\;i=1,...,C\\ x_{l2ss}=\frac{P_{a2}\left(P_{u,m},P_{l,m},x_{a1ss},I_{2}\right)}{\gamma_{x_{l2}}+k}+v_{2},\;\;m=1,...,A\\ N_{ss}=N_{\text{max}}\left(1-\frac{\gamma_{N}}{k}{x_{l2}}_{ss}\right)+v_{3} \end{array}\right)$$

for the genetic lysis circuit with activator-regulated promoter RBS component in Figure 1(b). In these equations, the Gaussian noise parameter *v_i_, i =*1, 2, with a zero mean and variance of *σ_i_*^2^, denotes cellular noise for both the transcriptional and translational gene expression processes in the steady state. *v*_3 _denotes cellular noise in cell population density in the steady state. In (5) and (6), the steady state population density *N_SS _*changes depending on inducer concentration, constitutive promoter-RBS components, repressor-regulated components, and activator-regulated promoter-RBS components, i.e. *i *= 1,...,*C, j *= 1,...,*R *or *i *= 1,...,*C, m *= 1,...,*A*. In this study, we select values for these components from promoter-RBS libraries (see Additional file 1) to achieve a specified I/O reponse with the genetic lysis circuit. The details of the construction procedure for promoter-RBS component libraries can see in Additional file 1.

In general, biological components are inherently uncertain in a molecular biological system. Hence, parameter uncertainties in equations (5) and (6) must be taken into consideration. For example, the kinetic parameters of the promoter-RBS components including the processes of transcription and translation, the degradation rates of regulatory proteins, dilution rates of the cells, and the lysis rates of the lysis proteins, are all stochastically uncertain *in vivo*, as a result of gene expression noise from biochemical processes, thermal fluctuations, DNA mutation, parameter estimation errors, and evolution [16]. These are defined as follows:

(7) $$\begin{array}{c} P_{u,i}\to P_{u,i}+\Delta P_{u,i}n_{1}\left(t\right),\;\\ P_{u,j}\to P_{u,j}+\Delta P_{u,j}n_{2}\left(t\right),\;P_{l,j}\to P_{l,j}+\Delta P_{l,j}n_{2}\left(t\right),\\ P_{u,m}\to P_{u,m}+\Delta P_{u,m}n_{2}\left(t\right),\;P_{l,m}\to P_{l,m}+\Delta P_{l,m}n_{2}\left(t\right),\\ \gamma_{xr1}\to \gamma_{xr1}+\Delta \gamma_{xr1}n_{1}\left(t\right),\;\gamma_{xa1}\to \gamma_{xa1}+\Delta \gamma_{xa1}n_{1}\left(t\right),\\ \gamma_{xl2}\to \gamma_{xl2}+\Delta \gamma_{xl2}n_{2}\left(t\right),\\ k\to k+\Delta kn_{3}\left(t\right),\\ \gamma_{N}\to \gamma_{N}+\Delta \gamma_{N}n_{3}\left(t\right) \end{array}$$

where Δ*P*_u,i_, Δ*P*_u,j_, Δ*P*_l,j_, Δ*P*_u,m_, Δ*P*_l,m_, Δ*γ*_xr1 _Δ*γ*_xa1_, Δ*γ*_xl2_, Δ*γ*_N_, and Δ*k *denote the standard deviations of the corresponding stochastic parameters, and *n_i_*(*t*), *i *= 1,2,3 denote Gaussian noise, have zero mean and unit variance, and account for random fluctuation sources. Thus, Δ*P*_u,i_, Δ*P*_u,j_, Δ*P*_l,j_, Δ*P*_u,m_, Δ*P*_l,m_, Δ*γ*_xr1_, Δ*γ*_xa1_, Δ*γ*_xl2_, Δ*γ*_N_, and Δ*k *denote the deterministic aspects of parameter variation, and *n*_i_(*t*), *i *= 1,2,3 denote the random fluctuation sources. The kinetic parameters in the steady state model in equations (5) and (6) are replaced by the parameter perturbations shown in (7) for robust design of the genetic circuit. These parameter fluctuations must be considered in the design procedure so that the synthetic genetic circuit can tolerate fluctuations *in vivo *[17].

### Design specifications for the genetic lysis circuit

The purpose of our design is to construct a genetic lysis circuit by selecting a set of suitable promoter-RBS components from the corresponding libraries in combination with a feasible range of inducer concentrations, to achieve optimal tracking of a desired I/O response. To achieve this, the following design specifications are needed:

- Desired I/O response *N_ref_*(*I*) of the genetic lysis circuit.

- Well-characterized promoter-RBS component libraries and a feasible range of inducer concentrations.

- Standard derivations of parameter fluctuations and environmental disturbances to be tolerated *in vivo*.

- A cost function between the steady state cell population density *N_SS _*in equations (5) and (6) and the desired reference steady state cell population density *N_ref _*as follows:

(8) $$J\left(S,I\right)=E\int {\left(N_{ss}\left(S,I\right)-N_{ref}\left(I\right)\right)}^{2}dI$$

where *S *denotes the set of promoter-RBS components *i *and *j *(or *i *and *m*) selected from the corresponding component libraries, i.e. *S *= (*i, j*) for constitutive promoter-RBS components and repressor-regulated promoter-RBS components in (5), and *S *= (*i, m*) for constitutive promoter-RBS components and activator-regulated promoter-RBS components in (6). The inducer concentration *I *for *I*_1 _is *S *= (*i, j*) and *I*_2 _is *S *= (*i, m*).

If the cost function in equation (8) is minimized by choosing the most appropriate set of components in combination with a feasible range of inducer concentrations under design specifications, the cell population density of a engineered genetic lysis circuit will optimally match the specified steady state cell population density *N_ref_*(*I*) under intrinsic parameter fluctuations and environmental disturbances. Although the cost function can be minimized by traditional conventional search methods, the combination of promoter-RBS components with inducer concentrations to minimize *J*(*S, I*) will require long computation times as well as trial-and-error experimentation when component libraries become large. Hence, the more effective and efficient genetic algorithm (GA) search method is proposed to save time in evaluating and selecting promoter-RBS components. Since genetic algorithm searches for the optimal solution, on basis of the maximum fitness, which is inversely proportional to the minimum error in (8), we need to define the fitness function as follows

(9) $$F\left(S,I\right)=\frac{1}{J\left(S,I\right)}$$

Therefore,

(10) $$\underset{\left(S,I\right)}{\text{max}}F\left(S,I\right)=\frac{1}{\underset{\left(S,I\right)}{\text{min}}J\left(S,I\right)}$$

### Summary of design procedure for the genetic lysis circuit

- Choose a species for cell population density control. In this study, we used *E. coli*.

- Provide user-defined design specifications for the genetic lysis circuit.

- Select an initial set *S *of promoter-RBS components and inducer concentrations.

- Calculate the fitness function *F*(*S, I*) in equation (9) for each set *S *of promoter-RBS components and inducer concentrations.

- Create an offspring set *S *using GA operators such as reproduction, crossover, and mutation.

- Make copies of possible solutions, on basis of their fitness.

- Swap values between two possible solutions.

- Randomly alter the value in a possible solution.

- Calculate the cost value of the new set *S *obtained by natural selection. Stop when the design goal is achieved or an acceptable solution is obtained. Otherwise, create the next generation and return to step 5.

## Results

### Genetic lysis circuit design example for cell population density control *in silico *and verification via experiment *in vivo*

**Figure 2:**
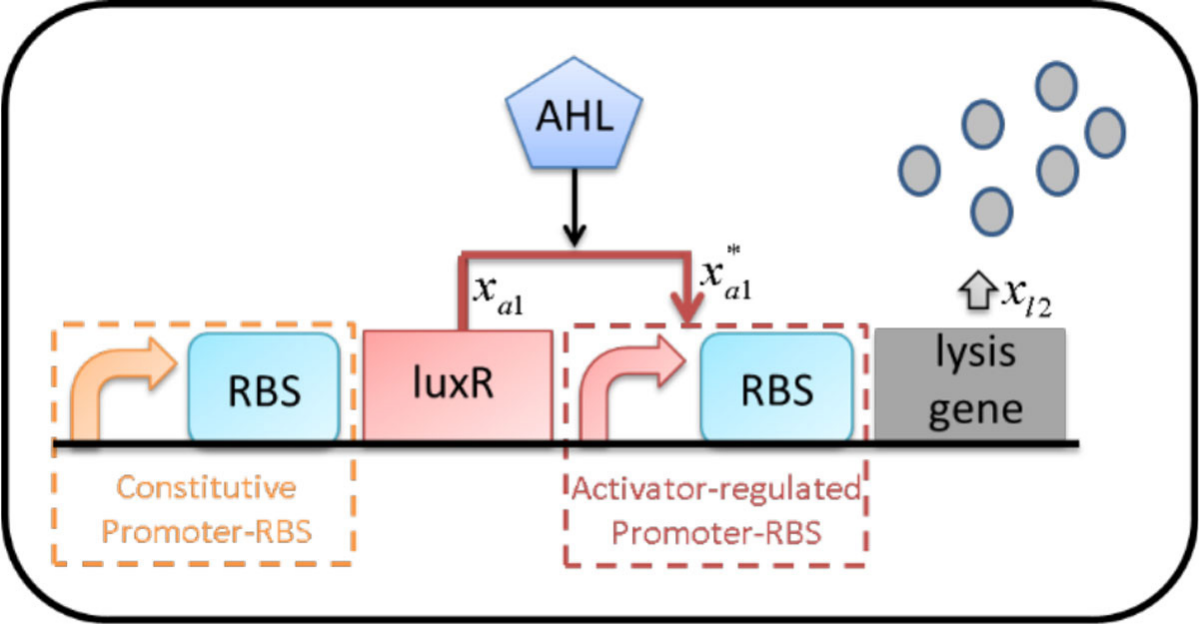
**Inducible LuxR-regulated circuit with lysis gene in *E***. *coli*. A constitutive promoter continuously produces the activator protein LuxR. The protein LuxR needs to form a complex with the inducer AHL. The LuxR-AHL complex constitutes a quorum sensing mechanism. It activates the downstream promoter and enhances expression of the lysis gene when this complex accumulates.

For the convenience of description and explanation, as shown in Figure 2, a genetic lysis circuit is assembled by selecting a set of promoter-RBS components, namely, a constitutive promoter-RBS component *C_i _*from Additional file 1 and an activator-regulated promoter-RBS component *A_m _*from Additional file 1. The lysis gene is embedded downstream of the activator-regulated promoter-RBS component. The genetic lysis circuit is divided into two stages. The first stage involves a constitutive promoter-RBS component *C_i _*for producing regulatory protein, LuxR. The second stage involves an activator-regulated promoter-RBS component *A_m _*for driving the expression of the lysis protein. The external inducer AHL is used to regulate the lysis activity. The desired population response *N_ref_*(*I*) to different inducer concentrations described as follows:

(11) $$N_{ref}\left(I\right)=0.1+\frac{0.6}{1+2\times I^{2}}$$

Note that the standard deviations of parameter fluctuations that are supposed to be tolerated *in vivo *are given by

(12) $$\begin{array}{c} \Delta P_{u,i}=0.05P_{u,i},\;\Delta P_{u,m}=0.05P_{u,m},\;\Delta P_{l,m}=0.05P_{l,m},\;\Delta \gamma_{xa1}=0.05\gamma_{xa1},\;\\ \Delta \gamma_{xl2}=0.05\gamma_{xl2},\;\Delta k=0.05k,\;\Delta \gamma_{N}=0.05\gamma_{N} \end{array}$$

as well as the environmental noise parameters *v*_1 _and *v*_2_, for transcription and translation processes, and *v*_3 _for cell population density, are all Gaussian, with zero mean and unit variance. In order to efficiently solve the constrained optimal matching design problem of the genetic lysis circuit, a GA-based library search method is employed to search a set *S *from corresponding libraries in Additional file 1 to minimize the following cost function:

(13) $$J\left(S,I\right)=E\int {\left(N_{ss}\left(S,I\right)-N_{ref}\left(I\right)\right)}^{2}dI,\;I\in \left[0.1,\;10\right]\;\left(nM\right)$$

**Figure 3:**
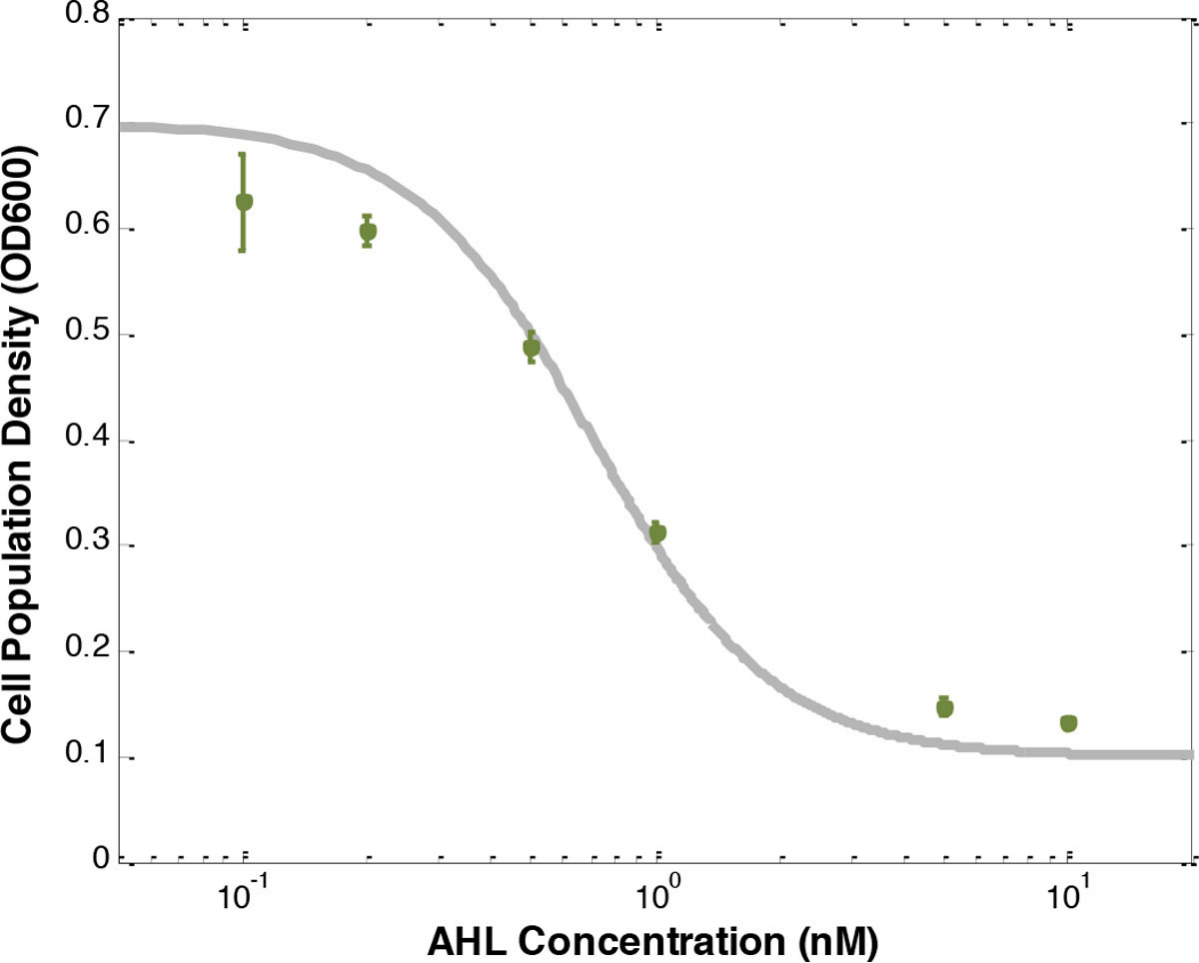
**The simulation and experiment results of a synthetic genetic lysis circuit**. By minimizing the cost function in (13) for the genetic lysis circuit in Figure 2, the adequate set *S*=(*C*_6_, *A*_12_) is selected from the corresponding libraries in Additional file 1. The green points are the experimental results by *S*=(*C*_6_,*A*_12_). The gray solid line is the desired I/O response.

**Figure 4:**
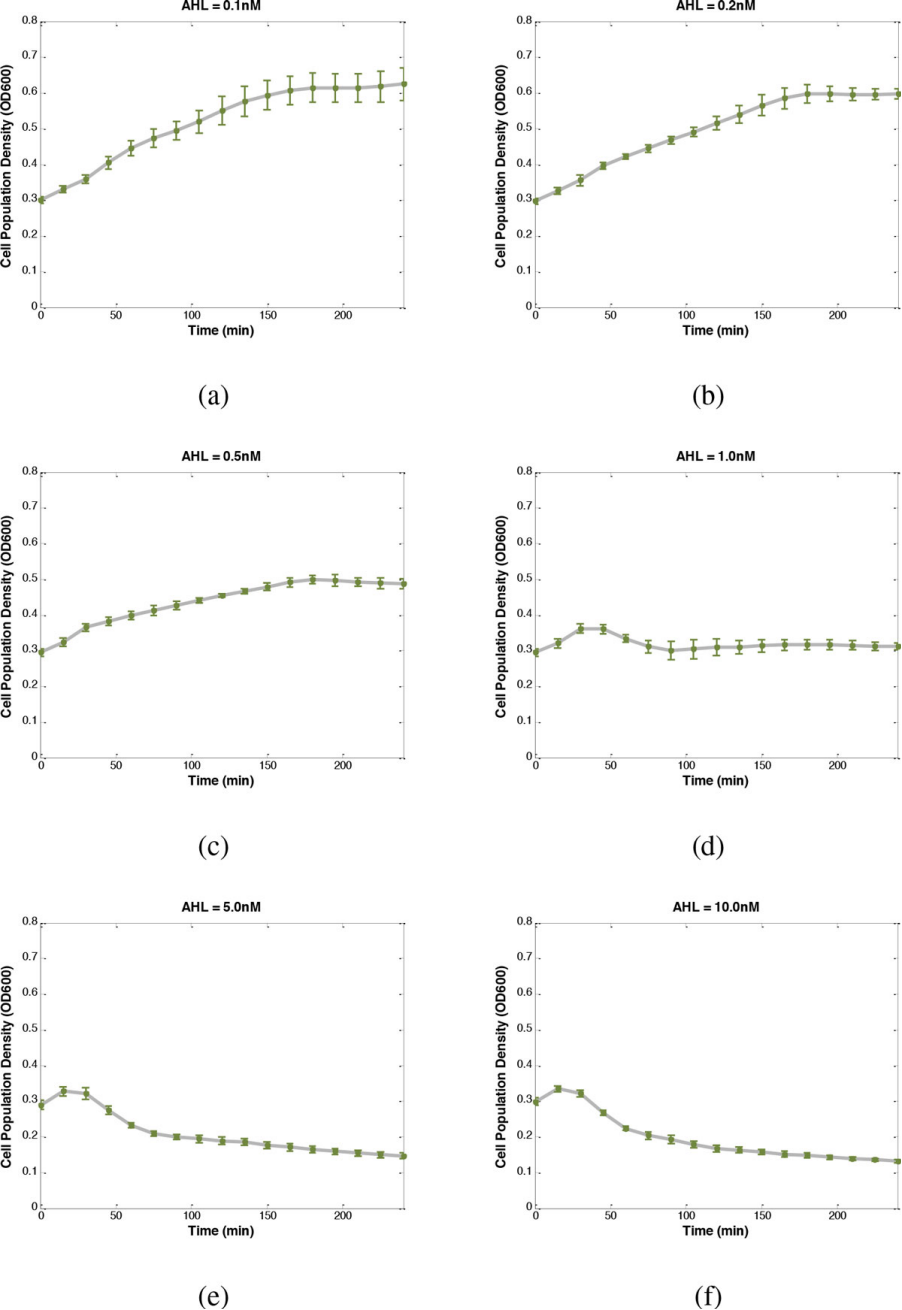
**Population density time profiles under different inducer concentrations of AHL**. The green points are the experimental results.

Then, to minimize the cost function (13), the adequate promoter-RBS components from the corresponding libraries are found to be *C*_6 _and *A_L2_*. The desired response is shown in Figure 3, with the experimental steady state O.D. 600 values taken from Figure 4 under different inducer concentrations at 240 min. Clearly, the cell population density of the genetic lysis circuit can robustly match the desired I/O response despite the intrinsic parameter fluctuations and environmental disturbances. If the desired cell density, for example, is 0.5, the suitable AHL concentration of 0.5nM can be taken, based on the prescribed I/O response. The experimental result of 0.487 is observed, with percentage error of 2.6%, confirming that the prescribed cell population density is well controlled by the proposed lysis circuit. Similarly, when the desired cell density is 0.3, the suitable AHL concentration of 1nM can be taken, based on the prescribed I/O response. The experimental result of 0.311 is observed, with percentage error of 3.6%, again indicating that the prescribed cell population density is well controlled by the proposed genetic circuit.

## Discussion

In this study, we focus on the design of synthetic genetic lysis circuit to achieve density control of cell population. A mathematical model is introduced to describe the dynamic and steady state regulatory behavior of the genetic lysis circuit. From the steady state mathematical models in (5) or (6), we find that if we want to control cell population density, we need to control lysis ability, which is related to transcriptional and translational processes. The promoter allows the RNA-polymerase molecules to latch onto a DNA strand and initialize the transcription of a downstream gene into mRNA, and the RBS allows the ribosome to bind and translate the mRNA. Hence, in this study, the promoter combined with the RBS is viewed as a genetic component for regulating lytic ability.

For convenient measurement of promoter-RBS components, we use GFP as a reporter to identify characterizations of promoter-RBS components. We can therefore use the parameters identified in Additional file 1 as component libraries to design the genetic lysis circuit. We also provide the design specifications for genetic lysis circuit for use by synthetic biologists. With a systematic approach, we can control the desired cell population density by selecting appropriate promoter-RBS component set in combination with a feasible range of inducer concentrations. In this study, for validation of the proposed design method, we perform some experiments with proper promoter-RBS components and inducer concentrations. The experimental results show that the simulation can robustly predict actual cell population density.

The precise control of cell population density with protein release is useful in medicine to cure disease[18], because changing medicine dosages may not achieve the desired effects. Additionally, evidence from systems biology indicates that apoptosis is involved in many pathways causing cell death [19,20]. Each pathway depends on expression levels and can trigger cell death. For example, we embed a genetic lysis circuit into *E. coli*. Through the control of different expression levels of repressor-regulated or activator-regulated promoter-RBS components, a simple genetic lysis circuit can regulate cell death. This bottom-up design approach can potentially be extended to other complicated processes involving programmed cell death, which could help us to understand systematic phenomena that have always existed in nature.

The construction procedure for cell population density control is very important for engineering a more complex synthetic genetic lysis circuit. We characterize the genetic circuit and provide the desired cell population density beforehand. Using GA, we search for an appropriate set of promoter-RBS components in combinations with a feasible range of inducer concentrations to achieve the desired cell population density. The GA provides a useful tool in the construction of genetic lysis circuit for control of cell population density. In future, when libraries are more complete, more effective and efficient design methods for synthetic genetic lysis circuits may aid developments in drug discovery, metabolic control, and therapeutic treatment.

## Conclusion

In this study, we engineer a genetic lysis circuit to control cell population density. Inducible promoter-RBS components are used to construct genetic lysis circuits with different lytic abilities for cell population control. Moreover, we provide four design specifications for cell population density control, allowing designers to select proper promoter-RBS components from libraries for the synthetic genetic lysis circuits. The design problem can be transformed by selecting proper promoter-RBS components in combination with a feasible range of inducer concentrations to achieve a desired I/O response. The proposed GA-based systematic searching methodology could provide synthetic biologists with a useful tool to design synthetic genetic lysis circuits. From the experimental results, we find that the data are close to the prescribed cell densities. Therefore, we believe that the proposed user-oriented design method for cell population density control will provide a useful guide in the rapidly growing field of synthetic biology.

## Competing interests

The authors declare that they have no competing interests.

## Authors' contributions

CYH developed the method, performed the analysis, evaluated the results, and wrote the manuscript. TCY participated in the method development and helped to draft the manuscript. LJL and RHH performed the experiments. BSC conceived of the study, provided essential guidance and revised the manuscript. All authors read and approved the final manuscript.

## Supplementary Material

Additional file 1 Supplementary appendix Click here for file

## Declarations

The full funding for the publication fee came from National Science Council of Taiwan.

This article has been published as part of *BMC systems Biology *Volume 8 Supplement 5, 2014: Proceedings of the 25th International Conference on Genome Informatics (GIW/ISCB-Asia): Systems Biology. The full contents of the supplement are available online at http://www.biomedcentral.com/bmcsystbiol/supplements/8/S5.
