# Supplementary material for: Systematic approach to Escherichia coli cell population control using a genetic lysis circuit
Source: BMC Syst Biol. 2014 Dec 12;8(Suppl 5):S7. doi: 10.1186/1752-0509-8-S5-S7 (PMC4305986; doi:10.1186/1752-0509-8-S5-S7)
Supplement: Additional file 1 — Supplementary appendix [file 1752-0509-8-S5-S7-S1.pdf]

Appendix

Appendix A: Construction of promoter-RBS components

The promoter allows the RNA-polymerase molecules to latch upon a DNA strand and initializes the transcription of downstream gene to mRNA, and the RBS allows the ribosomes to bind itself to perform the translation of mRNA. In this study, we suppose the effects of promoter on transcription and RBS on translation is integrated as a lumped reaction because the half-life of mRNA is shorter than the half-life of corresponding protein (typically, the half-life of mRNA of *E. coli* is 6.8 min [A1]). This consideration is similar to the results of previous literature [A2] despite the fact that there are a variety of strengths of both promoters and RBSs that could be combined to allow synthetic biologists to achieve a variety of transcription and translation strengths. Therefore, the combination of promoter and RBS is considered as a promoter-RBS component to regulate the expression of downstream gene. Meanwhile, the produced protein will be degraded by the corresponding protease and diluted by the cell growth. The protein expression driven by promoter-RBS component can be modeled as follows

$$\dot{x}(t) = P(P_u, P_l, TF, I) - (k + \gamma_x)x(t) \tag{A1}$$

where $x(t)$ denotes the concentration of protein, $P(P_u, P_l, TF, I)$ denotes the promoter-RBS regulation function of the maximum and minimum promoter-RBS strengths P_u and P_l from the corresponding components, *TF* denotes transcription factor concentration and *I* denotes inducer concentration. k and γ_x denote the dilution rate due to cell growth and degradation rate of corresponding protein, respectively.

There are three types of promoter-RBS components with three different promoter-RBS regulated functions i.e. constitutive, repressor and activator- regulated functions, respectively. The constitutive promoter-RBS component C_i without the impact by any TF can express its downstream gene continually at the maximum expression of the

component. So the promoter-RBS regulation function of constitutive promoter-RBS components i^{th} can be represented by

$$P_c(P_{u,i},0,0,0) = P_{u,i}$$

with the maximum strength $P_{u,i}$. Therefore, the protein expression of the constitutive promoter-RBS component set C_i in equation (A1) is modified as follows.

$$\dot{x}(t) = P_{u,i} - (k+\gamma_x)x(t) \quad , \quad i = 1,...,C \tag{A2}$$

where $x(t)$ is the concentration of target protein, the constitutive promoter-RBS regulation function only contains the maximum strength $P_{u,i}$, k is the dilution rate due to cell growth and γ_x is the degradation rate due to the cleavage of target protein $x(t)$ by protease. *i* denotes the ith constitutive promoter-RBS component of component set C_i.

The repressor-regulated promoter-RBS component R_j can be bound with free repressor to regulate its protein expression. The repressor-regulated promoter-RBS component, R_j, has the minimum and maximum strengths $P_{l,j}$ and $P_{u,j}$. The concentration of free repressor, $x_r^*(x_r,I_r)$, is regulated through the binding reaction by external inducer I_r as follows:

$$x_r^*(x_r,I_r) = \frac{x_r}{1+\dfrac{I_r}{K_{I_r}}} \tag{A3}$$

where x_r denotes the total repressor concentration including free and inducer-bound repressor, I_r denotes inducer concentration for regulating $x_r^*(x_r,I_r)$ and K_{I_r} denotes the inducer-repressor dissociation rate. The promoter-RBS regulation function

of repressor-regulated promoter-RBS component R_j can be represented as follows [A3]

$$P_r(P_{u,j}, P_{l,j}, x_r, I_r) = P_{l,j} + \frac{P_{u,j} - P_{l,j}}{1 + \left(\frac{x_r^*(x_r, I_r)}{K_r}\right)^{n_r}}$$

Therefore, the protein expression of repressor-regulation promoter-RBS component in (A1) is modified as the follows.

$$\dot{x}(t) = P_{l,j} + \frac{P_{u,j} - P_{l,j}}{1 + \left(\frac{x_r^*(x_r, I_r)}{K_r}\right)^{n_r}} - (k + \gamma_x)x(t) \quad , \quad j = 1, \ldots, R \qquad \text{(A4)}$$

where K_r and n_r denote the repressor-promoter binding affinity and binding cooperativity between the regulatory protein and the repressor-regulated promoter-RBS component, respectively; $P_{u,j}$ and $P_{l,j}$ denote the maximum and minimum strengths of repressor-regulated promoter-RBS of the jth component set, respectively. j denotes the jth repressor-regulated promoter-RBS component of component set R_j.

The activator-regulated promoter-RBS component A_m have the maximum and minimum strengths $P_{u,m}$ and $P_{l,m}$, respectively. The concentration of inducer-bound activator, $x_a^*(x_a, I_a)$, is regulated through the binding reaction by external inducer I_a as follows

$$x_a^*(x_a, I_a) = \frac{x_a \cdot I_a}{I_a + K_{I_a}} \qquad \text{(A5)}$$

where x_a denotes the total activator concentration including free and inducer-bound activator, I_a denotes inducer concentration for regulating the activator activity $x_a^*(x_a, I_a)$, and K_{I_a} denotes the inducer-activator dissociation rate. The regulatory

strength of activator-regulated promoter-RBS component A_m can be represented by a promoter-RBS regulation function as follows [A3]

$$P_a(P_{u,m}, P_{l,m}, x_a, I_a) = P_{l,m} + \frac{(P_{u,m} - P_{l,m}) \cdot \left(x_a^*(x_a, I_a)\right)^{n_a}}{\left(x_a^*(x_a, I_a)\right)^{n_a} + K_a^{\ n_a}}$$

Therefore, the protein expression of activator-regulated promoter-RBS component m^{th} in (A1) is modified as follows

$$\dot{x}(t) = P_{l,m} + \frac{(P_{u,m} - P_{l,m}) \cdot \left(x_a^*(x_a, I_a)\right)^{n_a}}{\left(x_a^*(x_a, I_a)\right)^{n_a} + K_a^{\ n_a}} - (k + \gamma_x)x(t) \quad , \quad m = 1, \ldots, A \qquad \text{(A6)}$$

where K_a and n_a denote the inducer binding affinity and binding cooperativity between regulatory protein and activator-regulated promoter-RBS component, respectively; $P_{u,m}$ and $P_{l,m}$ denote the maximum and minimum activator-regulated promoter-RBS strengths of activator-regulated promoter-RBS component of the mth component set, respectively. m denotes the mth activator-regulated promoter-RBS component of component set A_m.

We use the steady state GFP values to characterize promoter-RBS components by means of the nonlinear least square estimation method [A4]. The identification results are listed in Tables A1, A2 and A3, respectively.

A1. Selinger DW, Saxena RM, Cheung KJ, Church GM, Rosenow C: **Global RNA half-life analysis in Escherichia coli reveals positional patterns of transcript degradation**. *Genome research* 2003, **13**(2):216-223.

A2. Alper H, Fischer C, Nevoigt E, Stephanopoulos G: **Tuning genetic control through promoter engineering**. *Proceedings of the National Academy of Sciences of the United States of America* 2005, **102**(36):12678-12683.

A3. Alon U: **An introduction to systems biology: design principles of biological circuits**, vol. 10: Chapman & Hall/CRC; 2007.

A4. Johansson R: **System modeling & identification**: Prentice-Hall, Inc.; 1993.

Table A1 The constitutive promoter-RBS library.

**Index**	**BioBrick component**	P_u
C_1	J23101-B0031	94.520
C_2	J23101-B0032	65.892
C_3	J23101-B0034	237.000
C_4	J23105-B0031	14.493
C_5	J23105-B0032	6.512
C_6	J23105-B0034	28.586
C_7	J23106-B0031	22.011
C_8	J23106-B0032	12.193
C_9	J23106-B0034	58.781

Table A2 The repressor-regulated promoter-RBS library.

**Index**	**BioBrick component**	$\boldsymbol{P_u}$	$\boldsymbol{P_l}$	**Parameters**
R_{T1}	R0040-B0031	11.965	3.031	K_{atc} = 6.794 ng/ml
R_{T2}	R0040-B0032	8.740	3.381	K_{TetR} = 45.130 nM
R_{T3}	R0040-B0034	162.557	4.837	n_{TetR} = 2

Table A3 The activator-regulated promoter-RBS library.

**Index**	**BioBrick component**	$\boldsymbol{P_u}$	$\boldsymbol{P_l}$	**Parameters**
A_{L1}	R0062-B0032	360.761	7.970	K_{AHL} = 4.541nM<br>K_{LuxR} = 55.964nM<br>n_{LuxR} = 1
A_{L2}	R0062-B0034	460.353	8.978	
